# Supplementary material for: Proteomic assessment of SKBR3/HER2+ breast cancer cellular response to Lapatinib and investigational Ipatasertib kinase inhibitors
Source: Front Pharmacol. 2024 Aug 29;15:1413818. doi: 10.3389/fphar.2024.1413818 (PMC11391243; doi:10.3389/fphar.2024.1413818)

Supplemental file 2

PSM detection reproducibility. Scatter plots representing the reproducibility of PSMs in three biological replicates of the nuclear and cytoplasmic fractions for each drug treatment experiment.

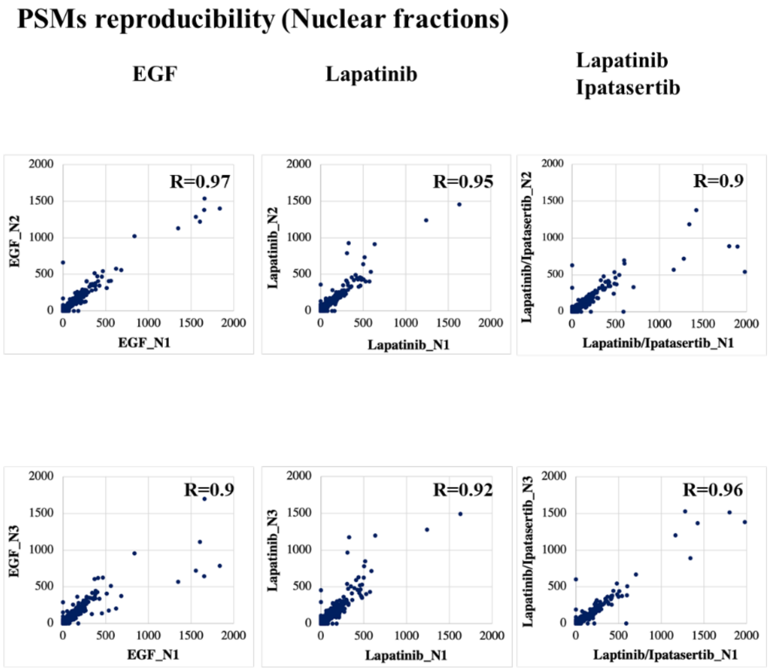

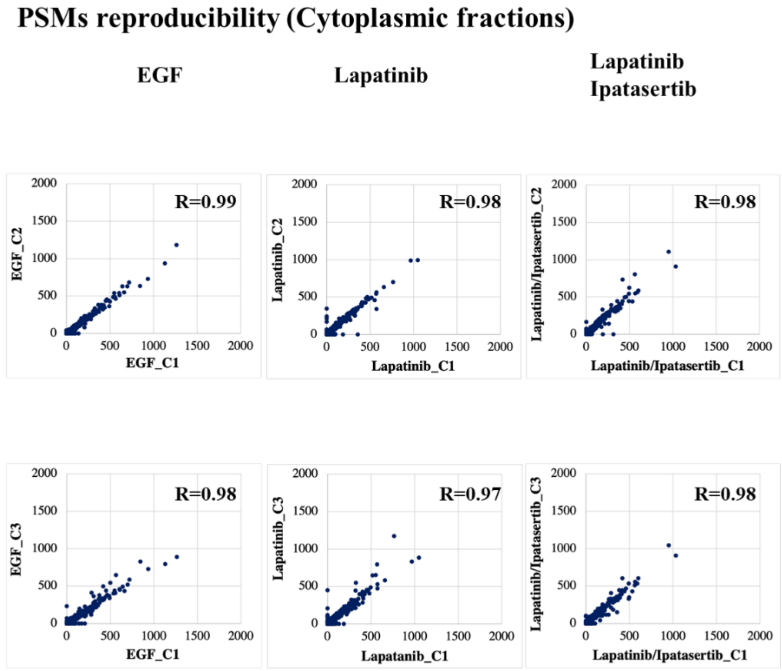

Peptide detection reproducibility. Scatter plots representing the reproducibility of peptide elution times and XCorr scores from each biological replicate of the cytoplasmic and nuclear fractions: replicate 1 (X-axis), replicate 2 (Y-axis), and replicate 3 (color bar).

Peptide reproducibility (XCorr)

EGF **Lapatinib** **Lapatinib Ipatasertib**

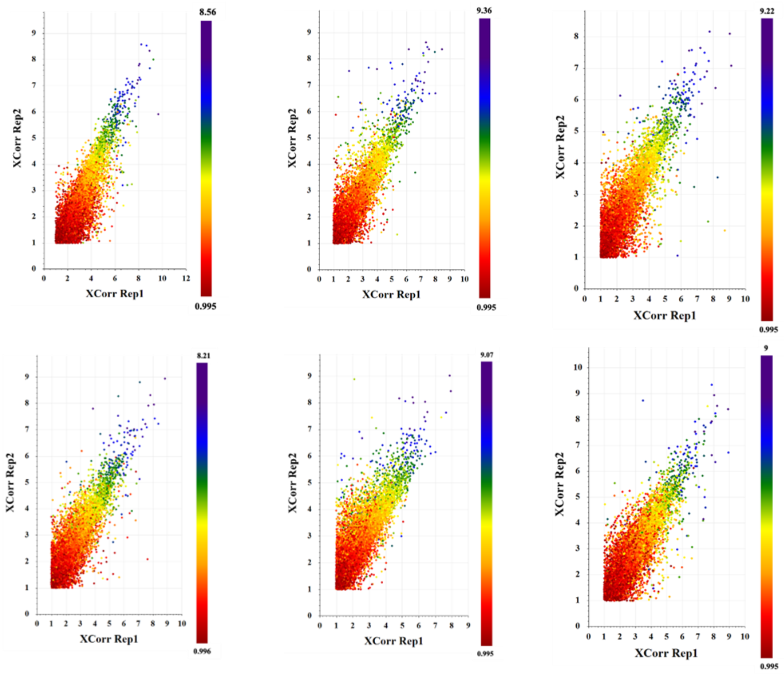

Protein differential expression. Volcano plots representing protein abundance measurements in the nuclear and cytoplasmic fractions of drug-treated Lapatinib/Ipatasertib vs Lapatinib-control cells. Differentially expressed proteins are indicated in red (up-regulated) and blue (down-regulated), displaying ≥2-fold change in abundance (p-value ≤0.05)

Lapatinib & Ipatasertib vs Lapatinib
Cytoplasmic fractions

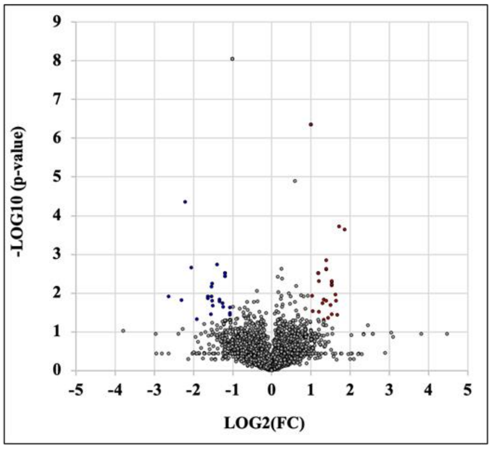

Lapatinib & Ipatasertib vs Lapatinib
Nuclear fractions

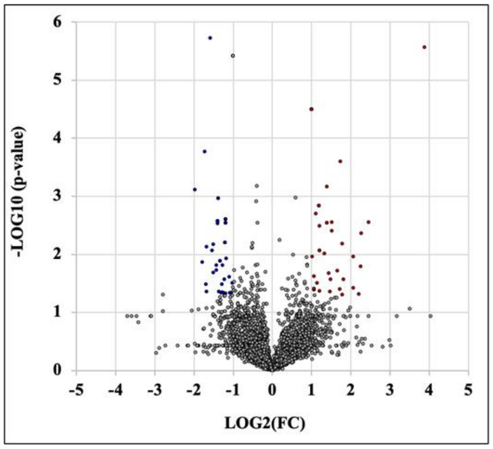

Supplement: Supplementary file 1 [file DataSheet1.zip › Supplemental file 2.PDF]
