# Supplementary material for: Proteomic assessment of SKBR3/HER2+ breast cancer cellular response to Lapatinib and investigational Ipatasertib kinase inhibitors
Source: Front Pharmacol. 2024 Aug 29;15:1413818. doi: 10.3389/fphar.2024.1413818 (PMC11391243; doi:10.3389/fphar.2024.1413818)

Supplemental file 6

Parallel Reaction Monitoring (PRM)/MS validation of selected proteins that changed expression level in response to the drug treatments.

Upregulated in Lapatinib

Serine-protein kinase ATM (ATM)
DIVDGMGITGVEGVFR, Charge +2, m/z = 832.9193 Da

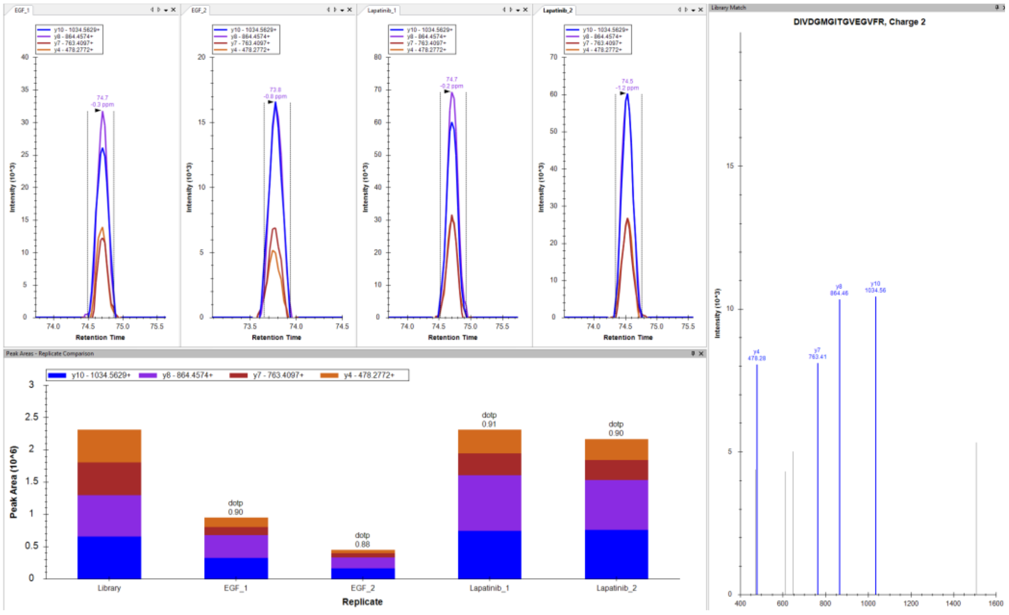

Serine-protein kinase ATM (ATM)
MTTDPMLAYLQPFR, Charge +2, m/z = 842.4153 Da

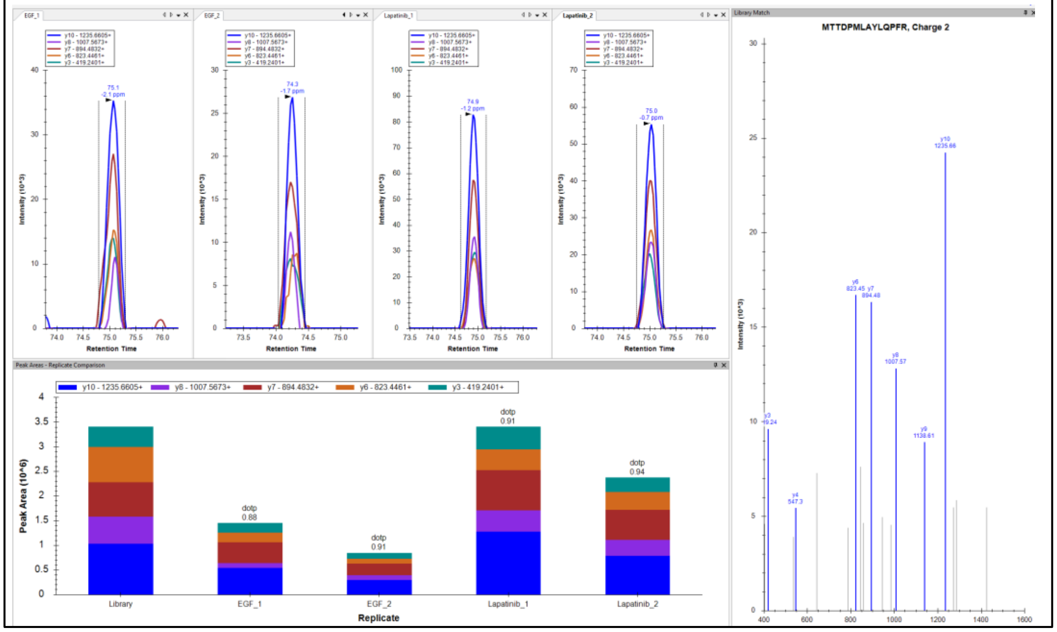

Nuclear mitotic apparatus protein 1 (NUMA1)
EFASHLQQLQDALNELTEEHSK, Charge +3, m/z = 856.4173 Da

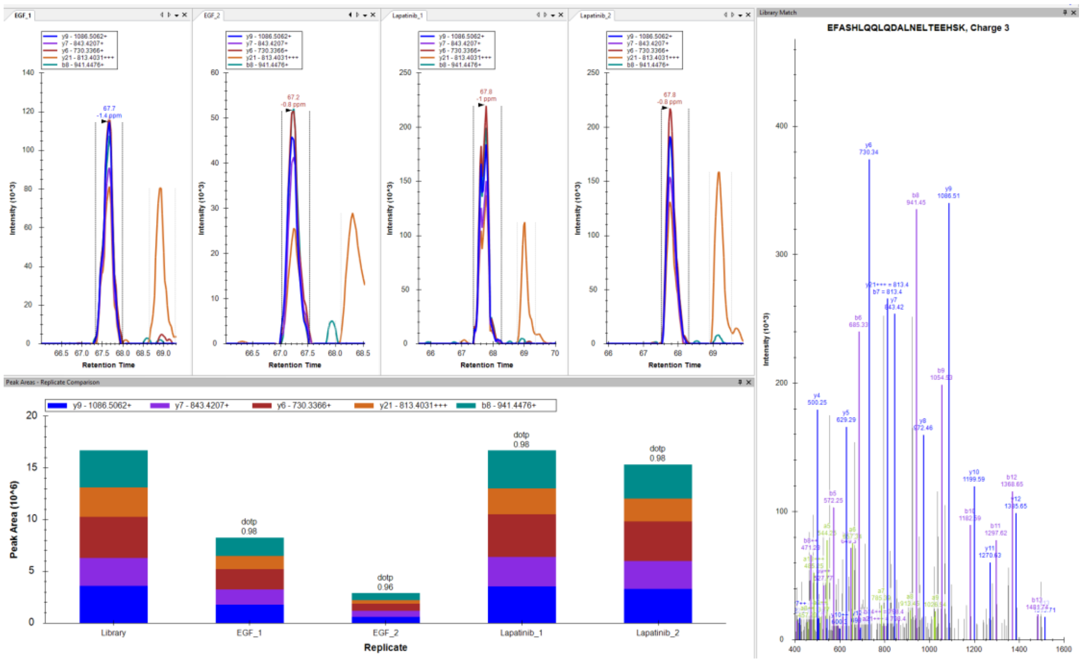

Nuclear mitotic apparatus protein 1 (NUMA1)
AQELGHSQSALASAQR, Charge +2, m/z = 827.4188 Da

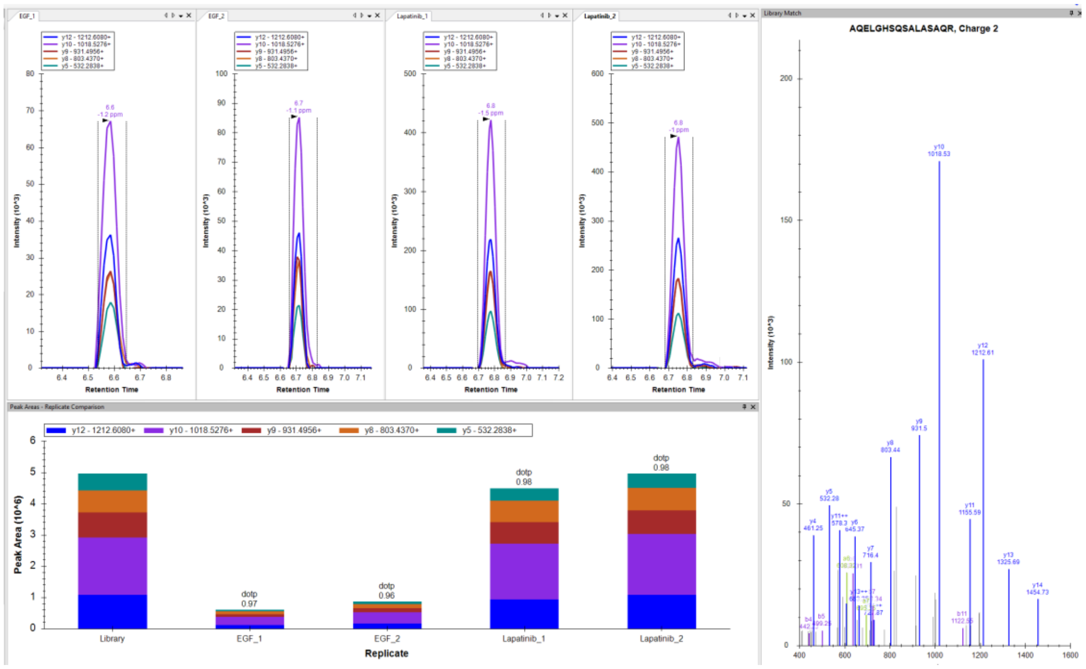

Programmed cell death protein 4 (PDCD4)

APQLVGQFIAR, Charge +2, m/z = 600.3484 Da

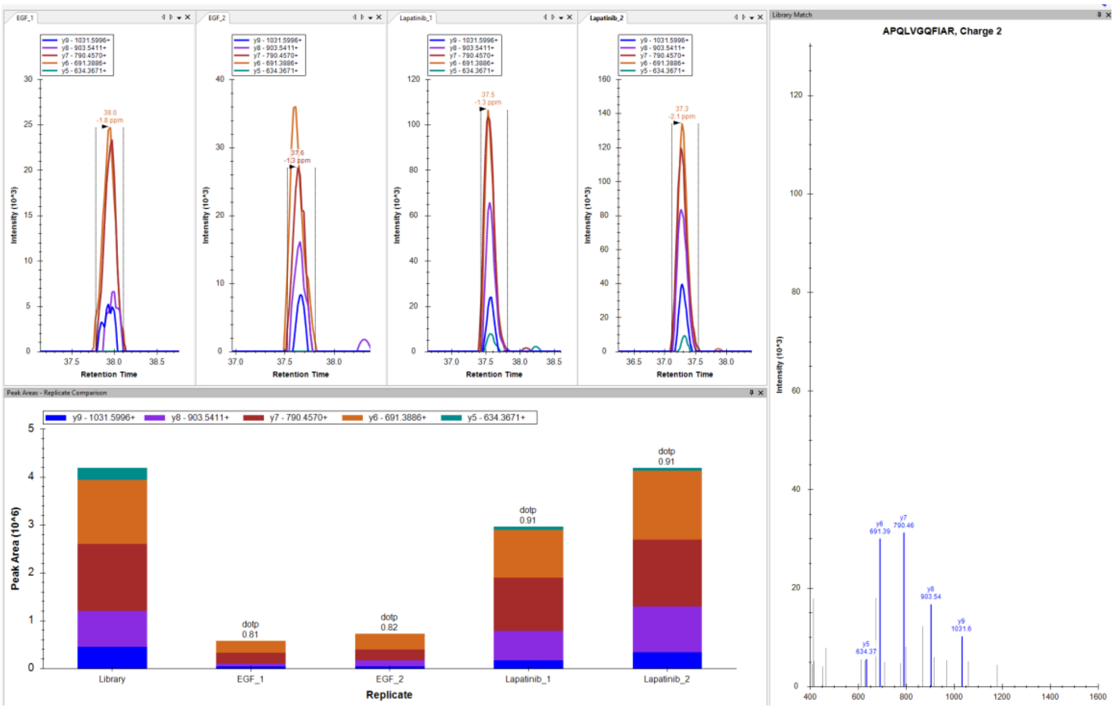

Programmed cell death protein 4 (PDCD4)

SGVPVLAVSLALEGK, Charge +2, m/z = 720.4270 Da

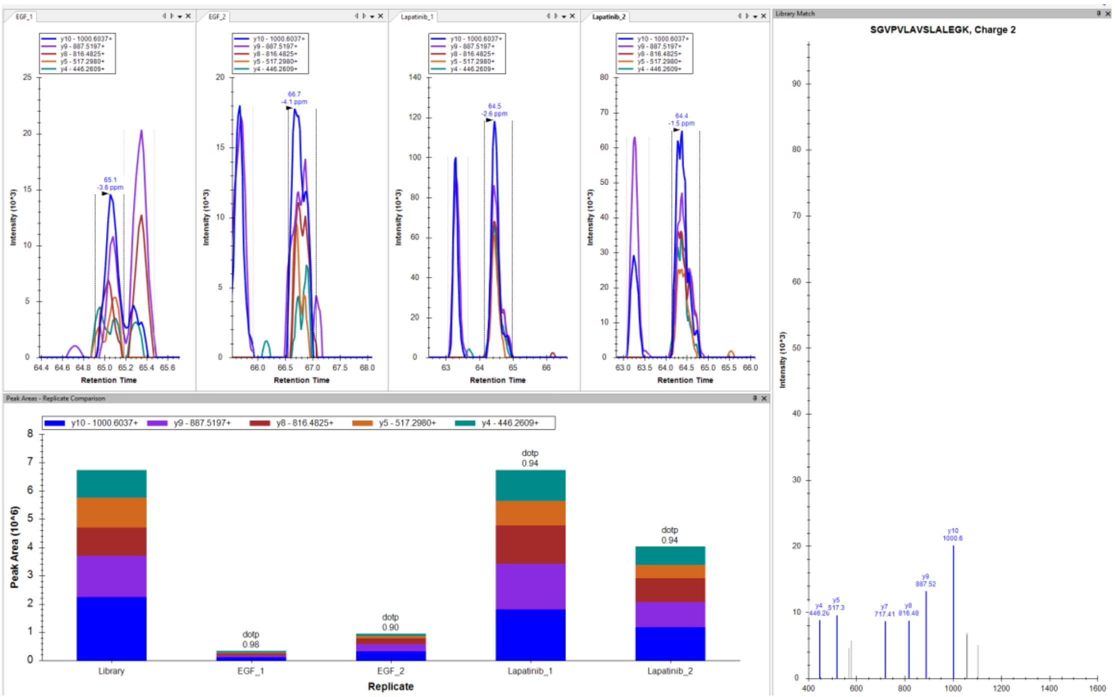

Downregulated in Lapatinib

DNA topoisomerase 2-alpha (TOP2A)
VTIDPENNLISIWNNGK, Charge +3, m/z = 643.0022 Da

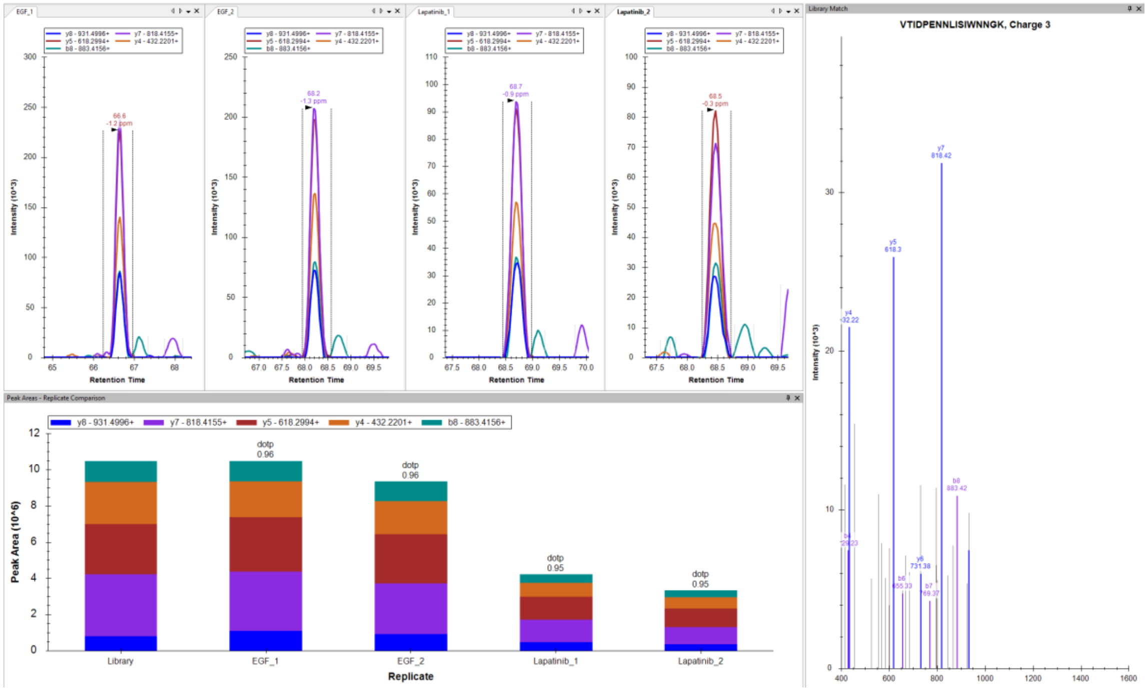

DNA topoisomerase 2-alpha (TOP2A)
EDLATFIEELEAVEAK, Charge +2, m/z = 903.9540 Da

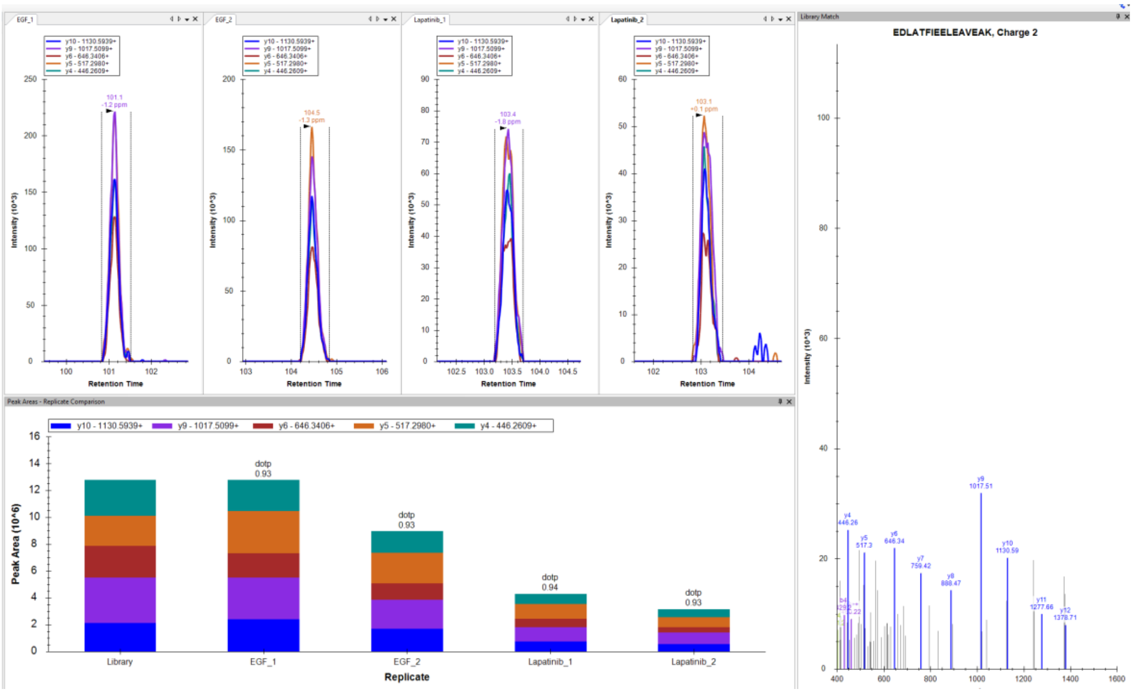

Proliferation marker protein Ki-67 (MKI67)
AVGASFPLYEPAK, Charge +2, m/z = 675.3586 Da

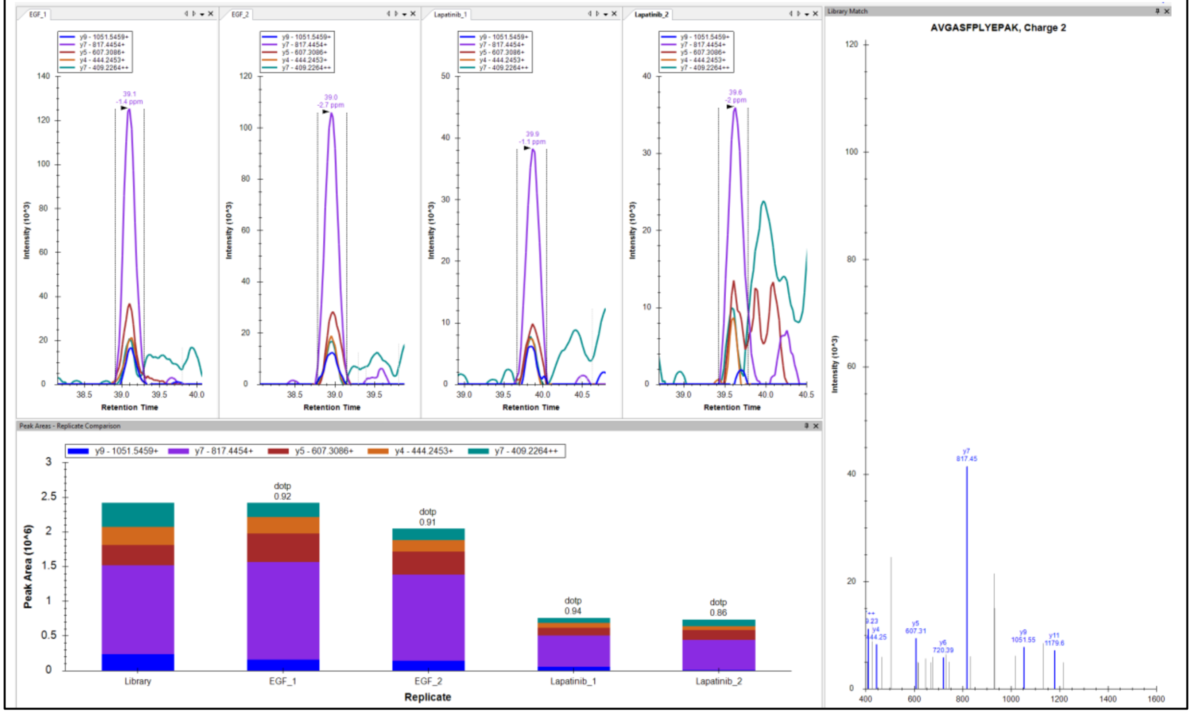

Proliferation marker protein Ki-67 (MKI67)
AQALEDLAGFK, Charge +2, m/z = 581.8088 Da

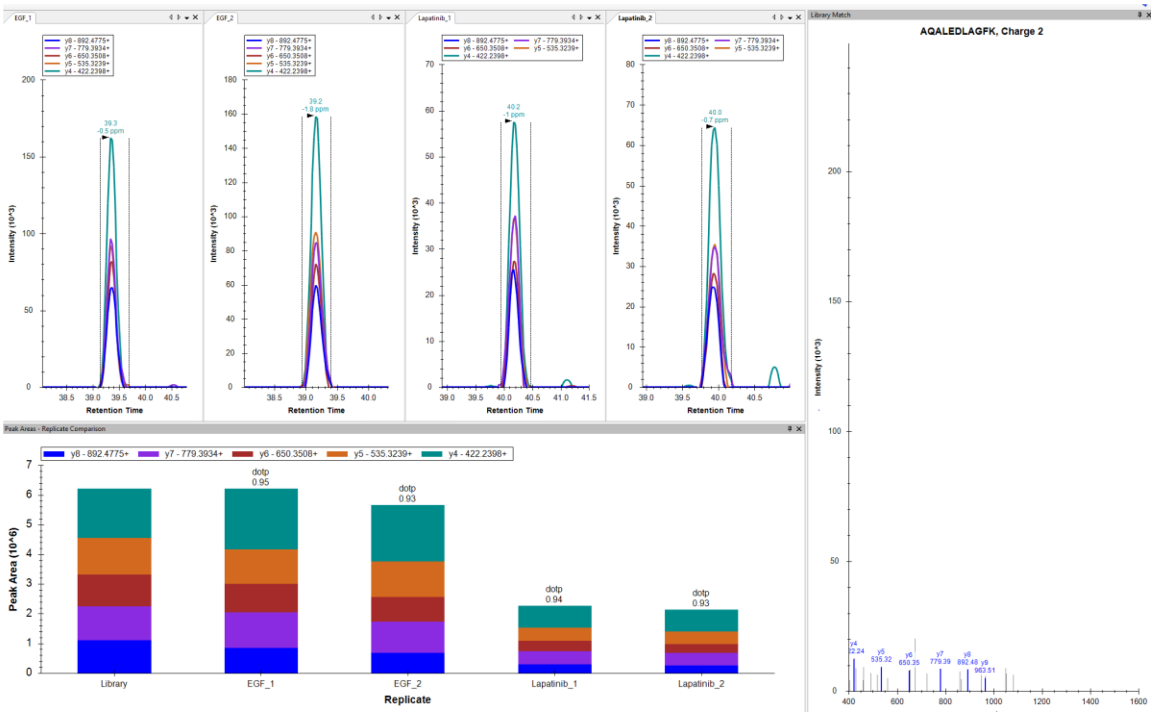

14-3-3 protein sigma (SFN)

VETELQGVCDTVLGLLDSHLIK, Charge +3, m/z = 794.7577 Da

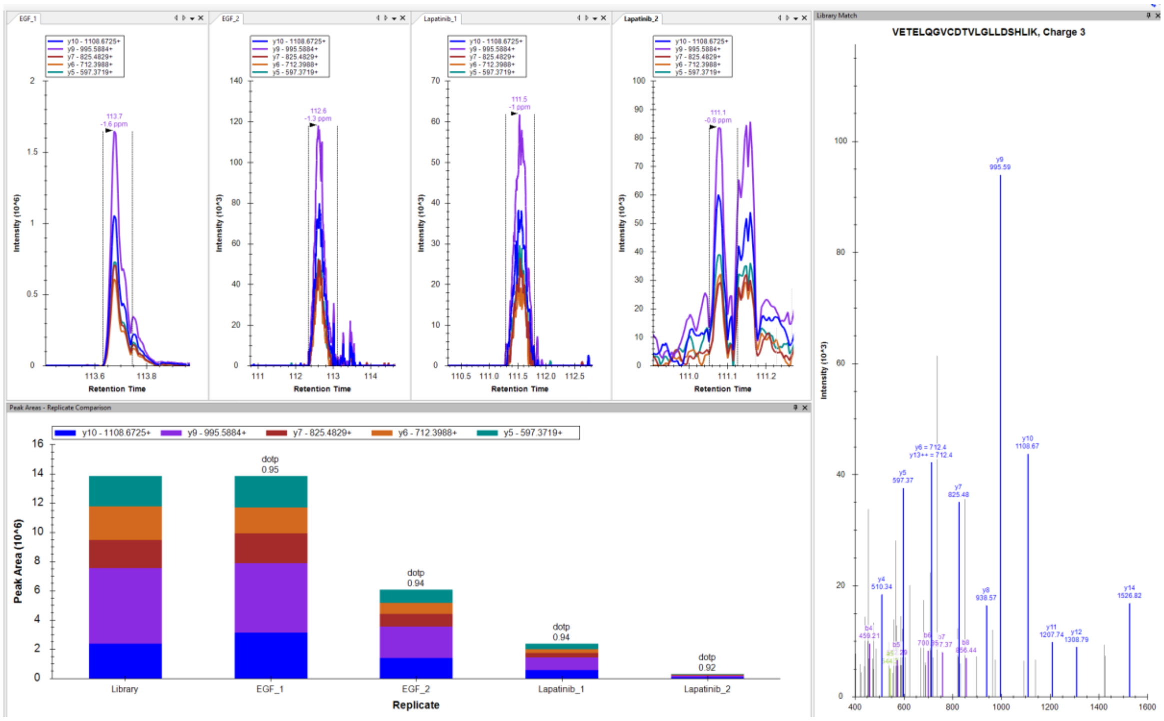

CD44 antigen (CD44)

YGFIEGHVVIPR, Charge +3, m/z = 462.9225 Da

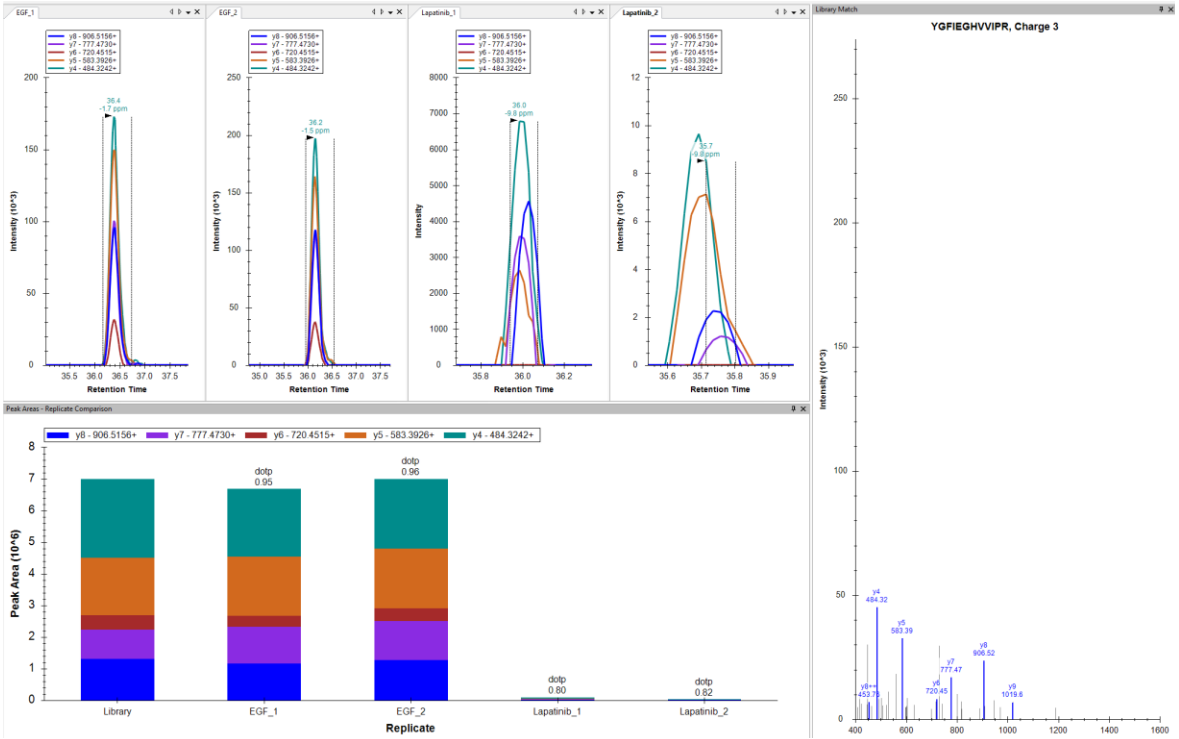

Upregulated in Lapatinib/Ipatasertib

Serine-protein kinase ATM (ATM)
MTTDPMLAYLQPFR, Charge +2, m/z = 842.4153 Da

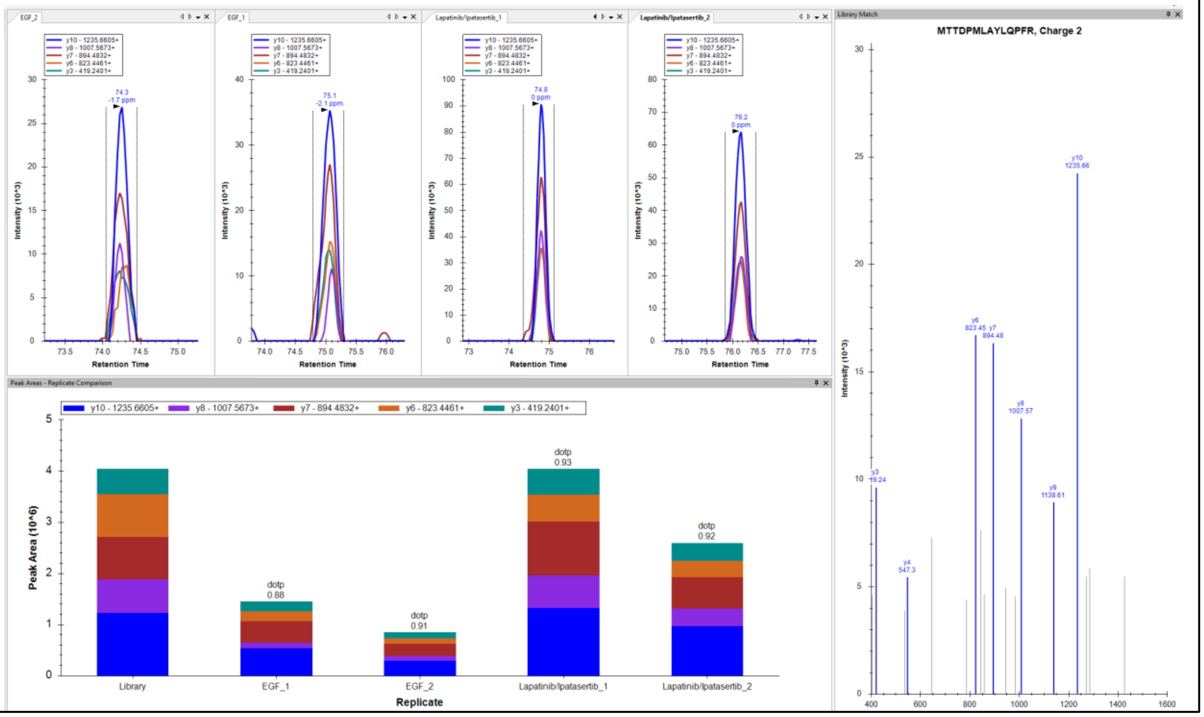

Serine-protein kinase ATM (ATM)
DIVDGMGITGVEGVFR, Charge +2, m/z = 832.9193 Da

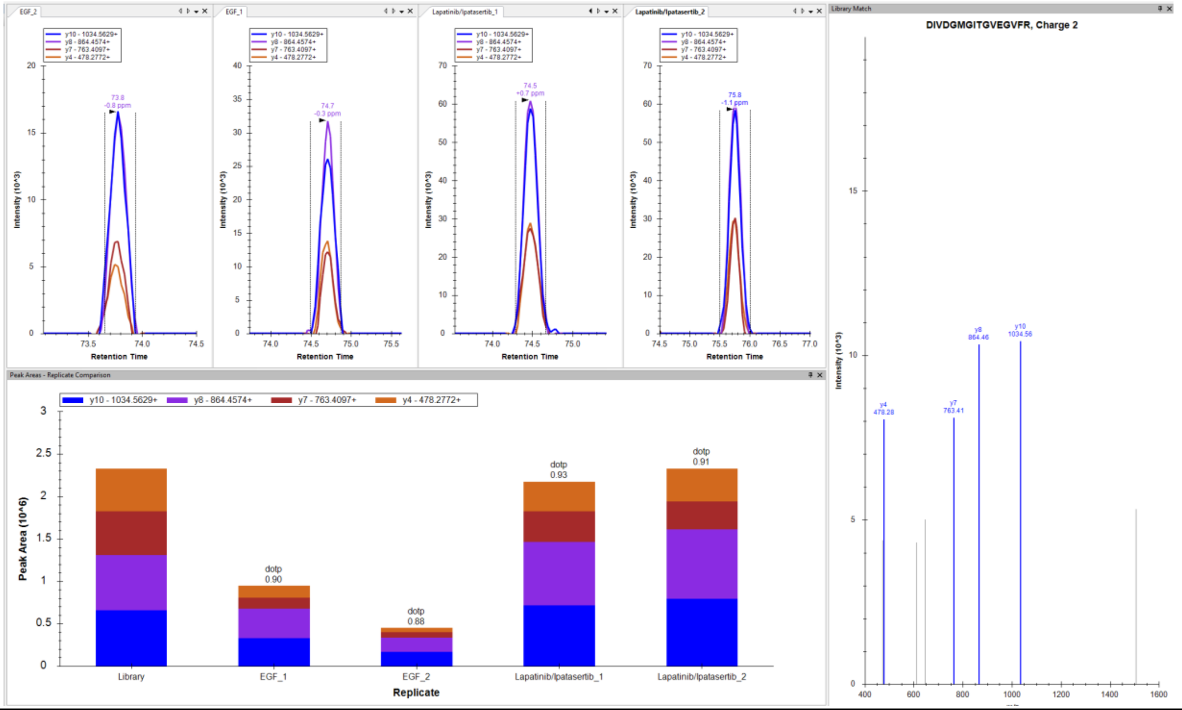

Nuclear mitotic apparatus protein 1 (NUMA1)
ASMQPIQIAEGTGITTR, Charge +2, m/z = 887.4618 Da

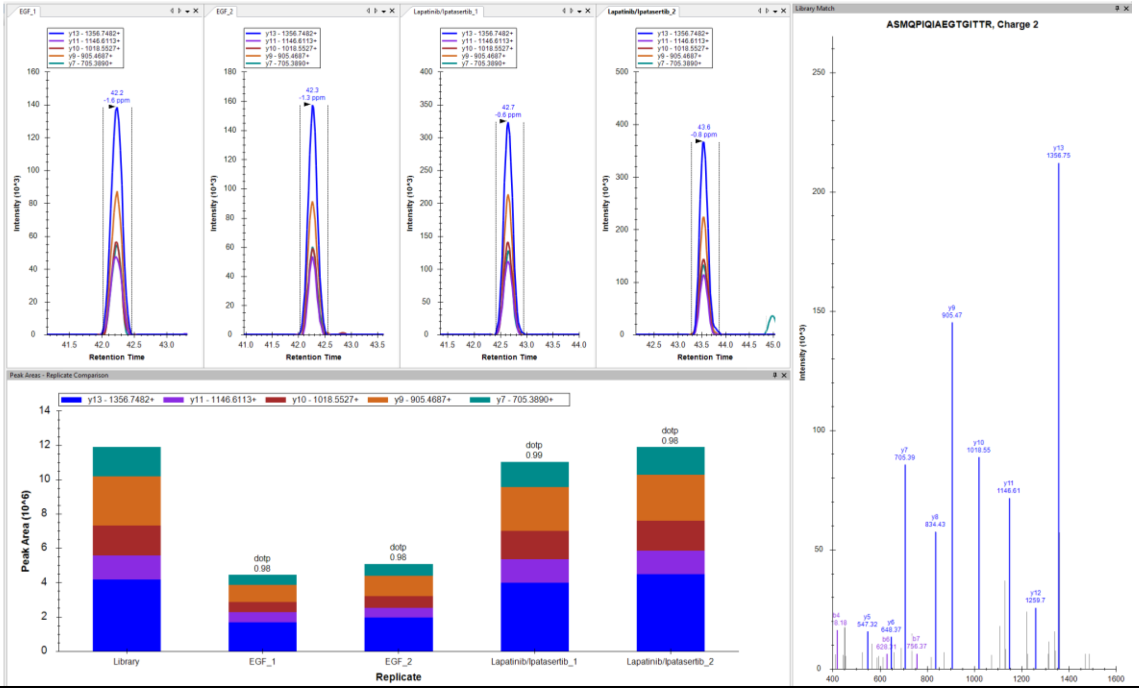

Nuclear mitotic apparatus protein 1 (NUMA1)
EFASHLQQLQDALNELTEEHSK, Charge +3, m/z = 856.4173 Da

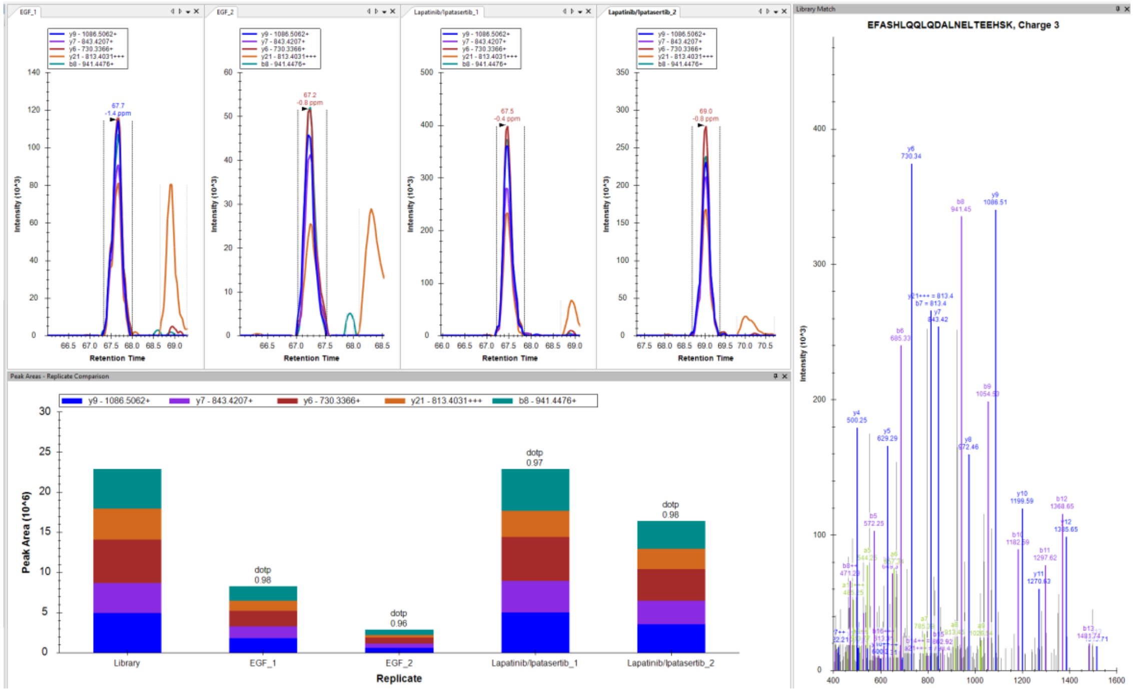

Programmed cell death protein 4 (PDCD4)

SGVPVLAVSLALEGK, Charge +2, m/z = 720.4270 Da

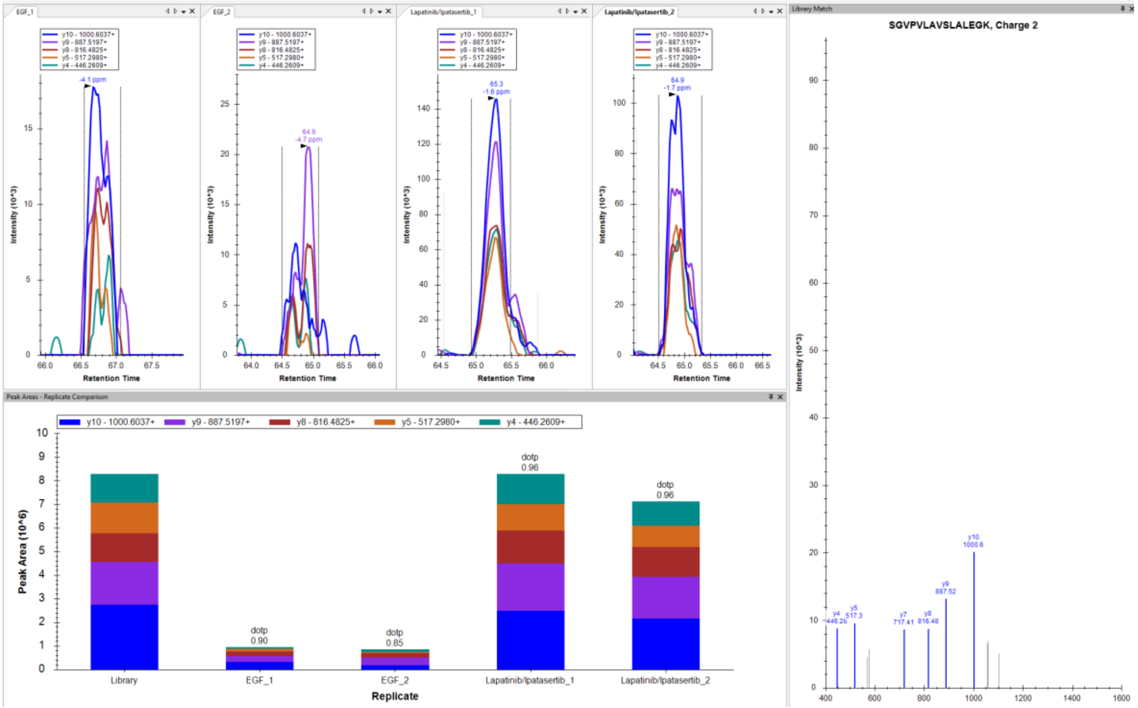

Programmed cell death protein 4 (PDCD4)

IYNEIPDINLDVPHSYSVLER, Charge +3, m/z = 829.4236 Da

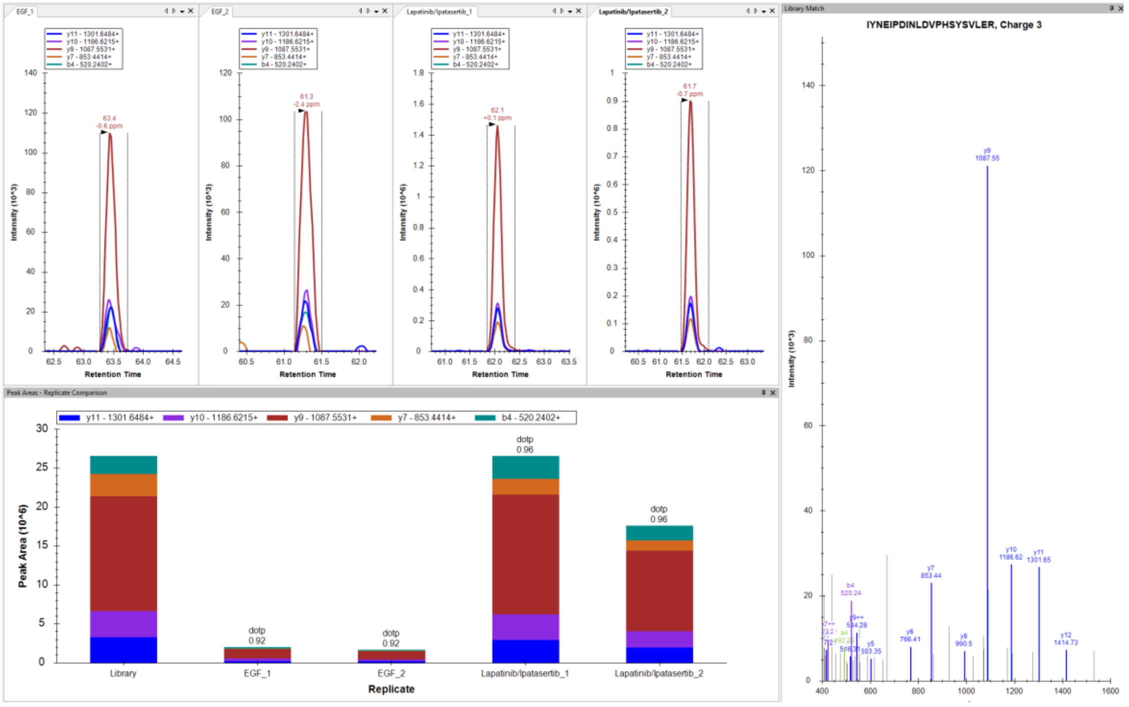

V-set domain-containing T-cell activation inhibitor 1 (VTCN1)

NVQLTDAGTYK, Charge +2, m/z = 605.3091 Da

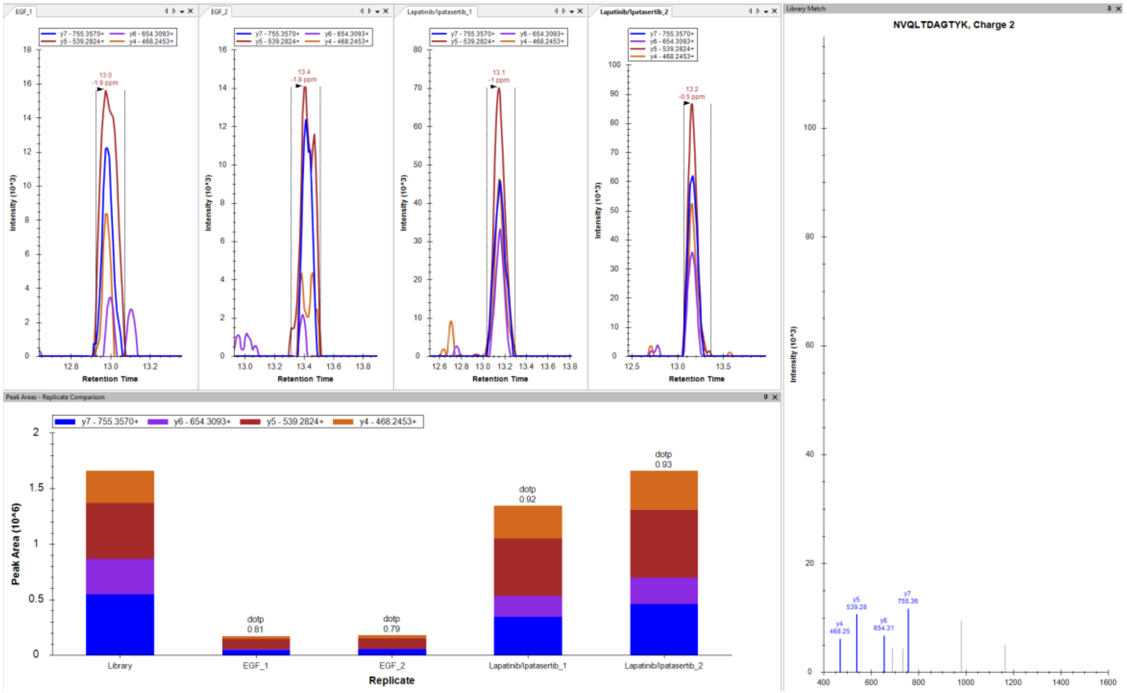

V-set domain-containing T-cell activation inhibitor 1 (VTCN1)

LSDIVIQWLK, Charge +2, m/z = 607.8608 Da

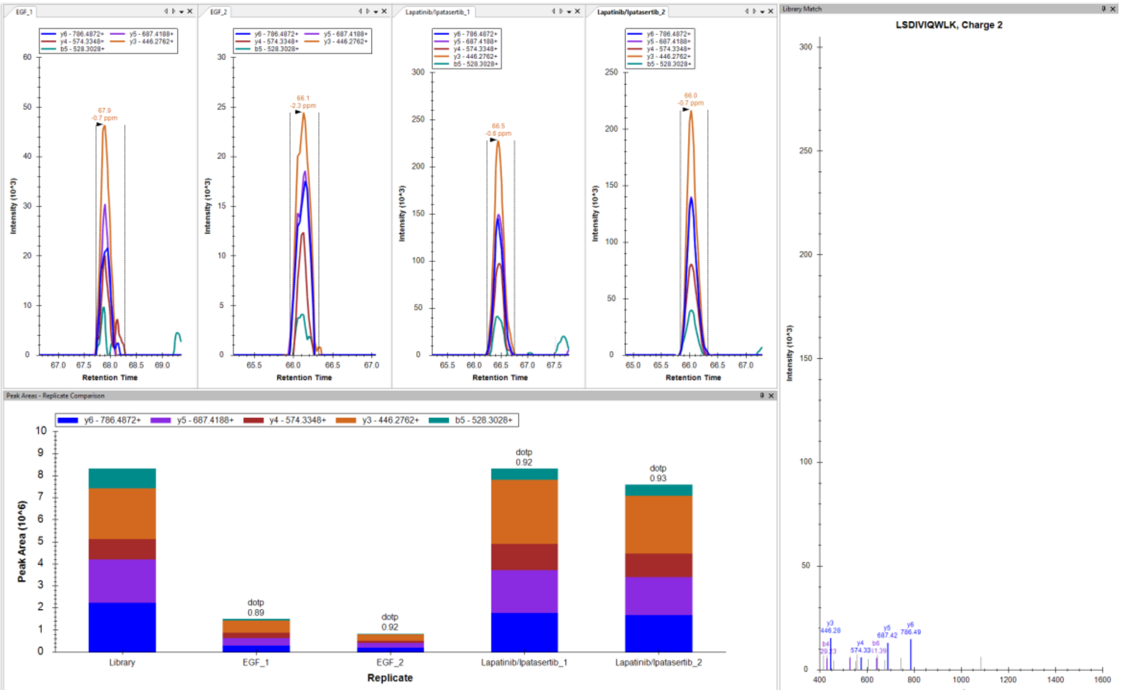

Downregulated in Lapatinib/Ipatasertib

DNA topoisomerase 2-alpha (TOP2A)
VTIDPENNLISIWNNGK, Charge +3, m/z = 643.0022 Da

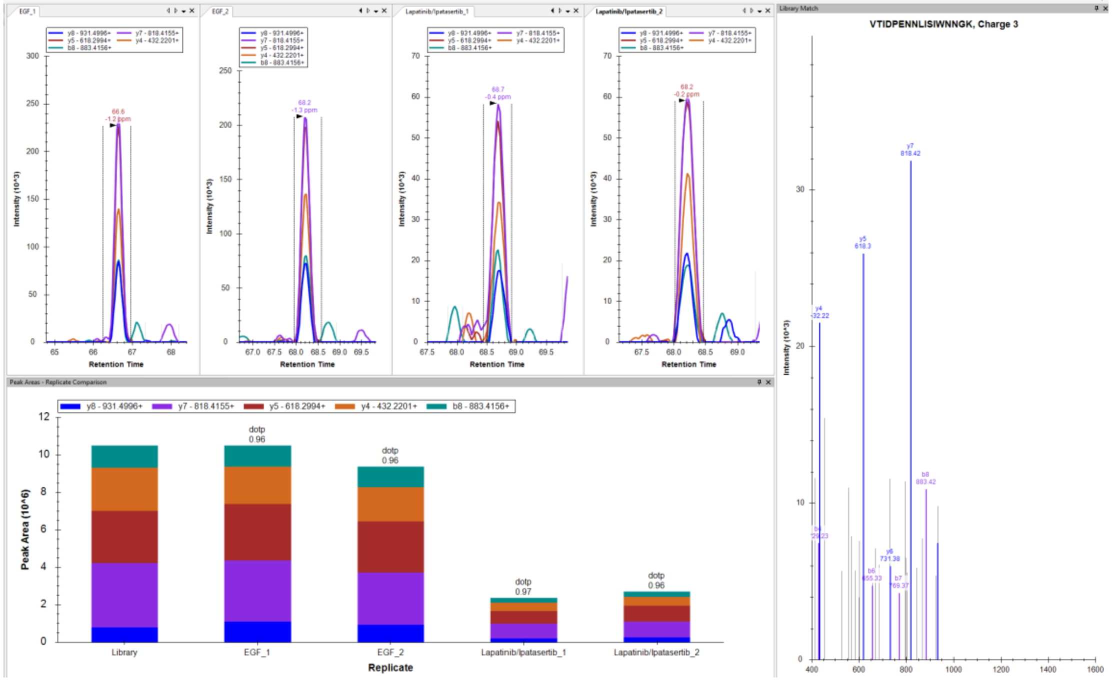

DNA topoisomerase 2-alpha (TOP2A)
EDLATFIEELEAVEAK, Charge +2, m/z = 903.9540 Da

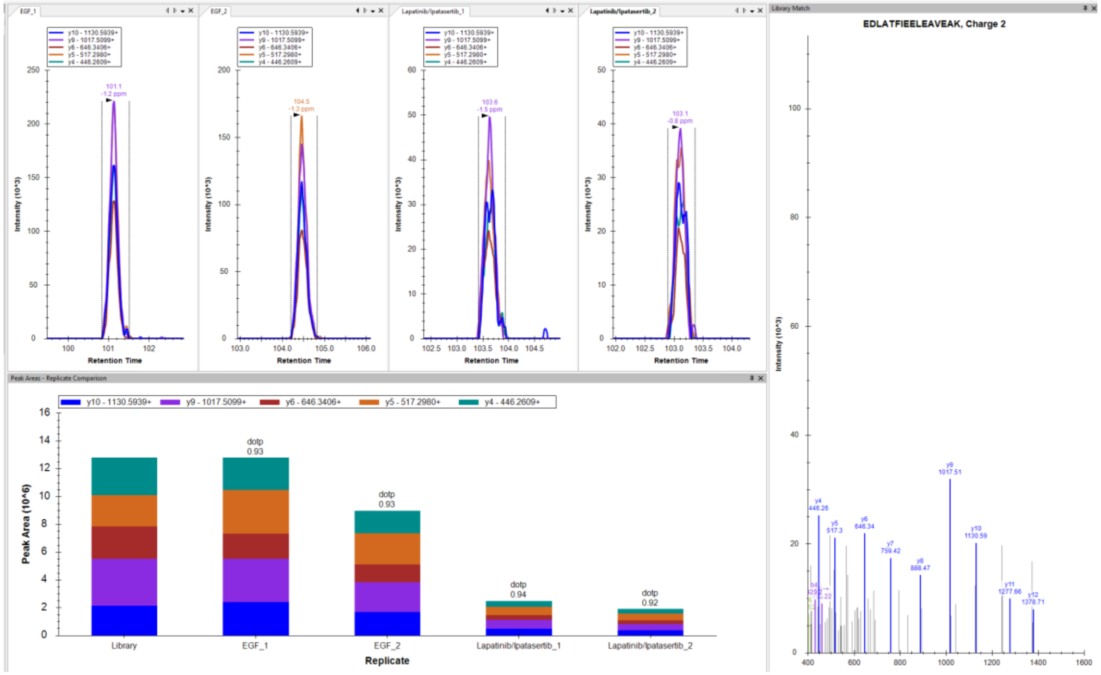

Proliferation marker protein Ki-67 (MKI67)

AQALEDLAGFK, Charge +2, m/z = 581.8088 Da

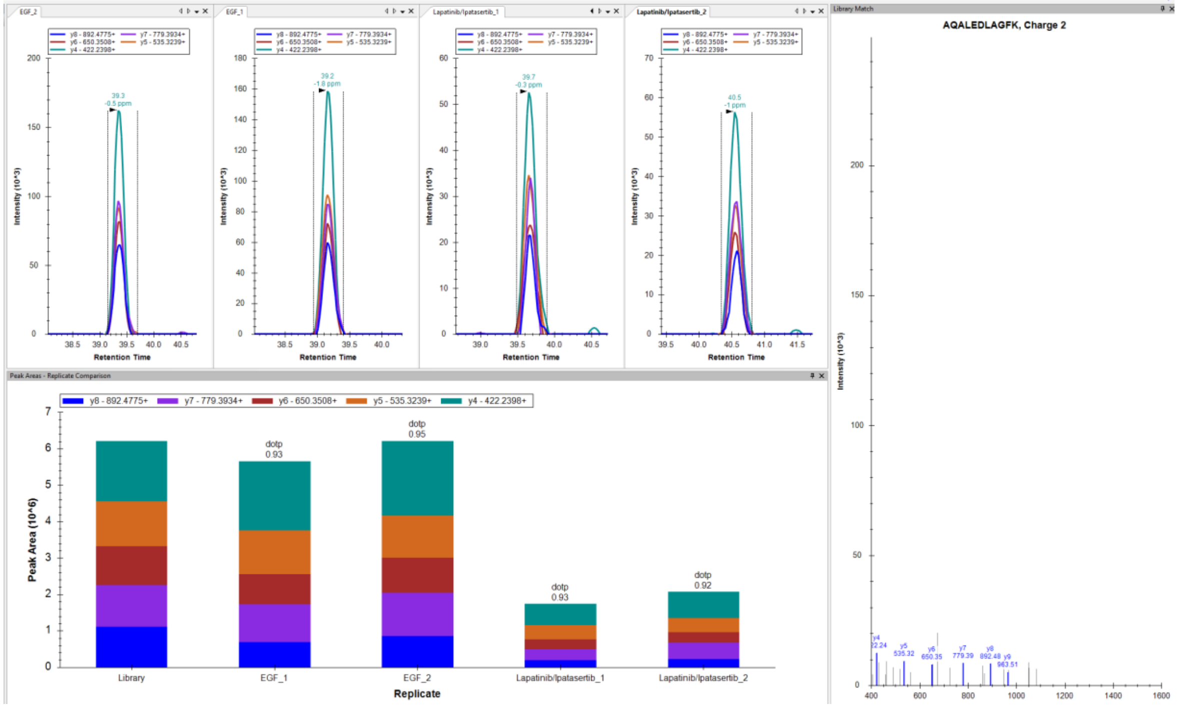

Proliferation marker protein Ki-67 (MKI67)

AVGASFPLYEPAK, Charge +2, m/z = 675.3586 Da

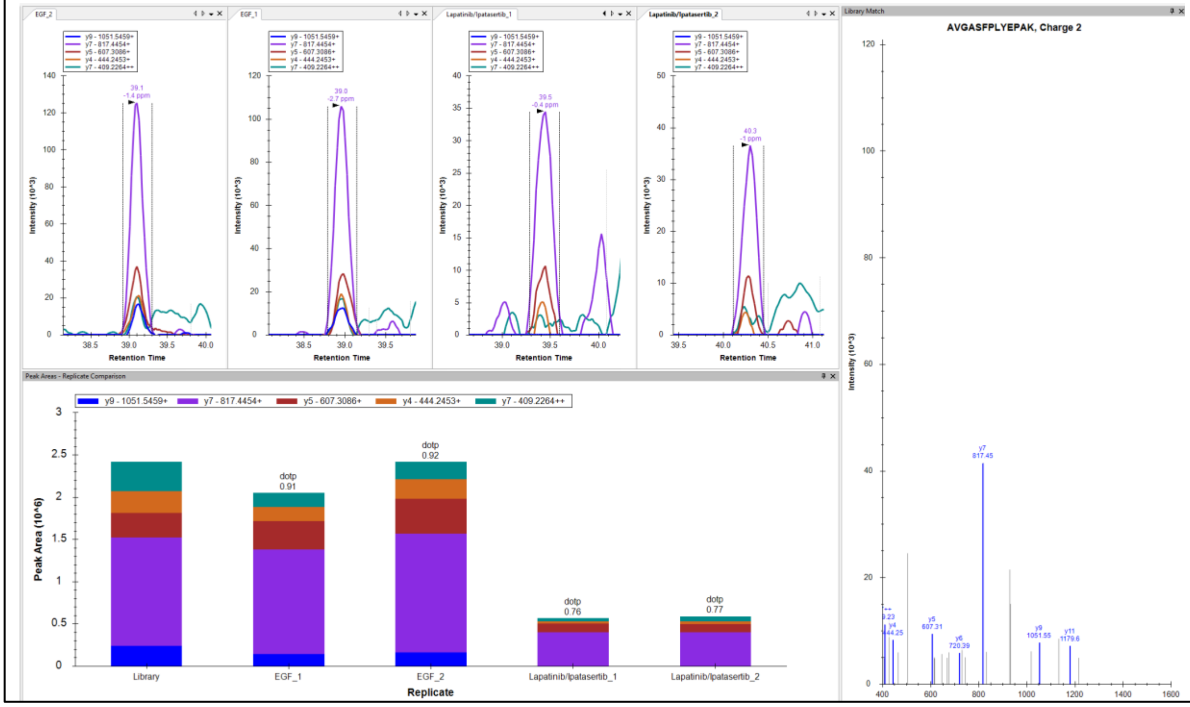

14-3-3 protein sigma (SFN)

VETELQGVCDTVLGLLDSHLIK, Charge +3, m/z = 794.7577 Da

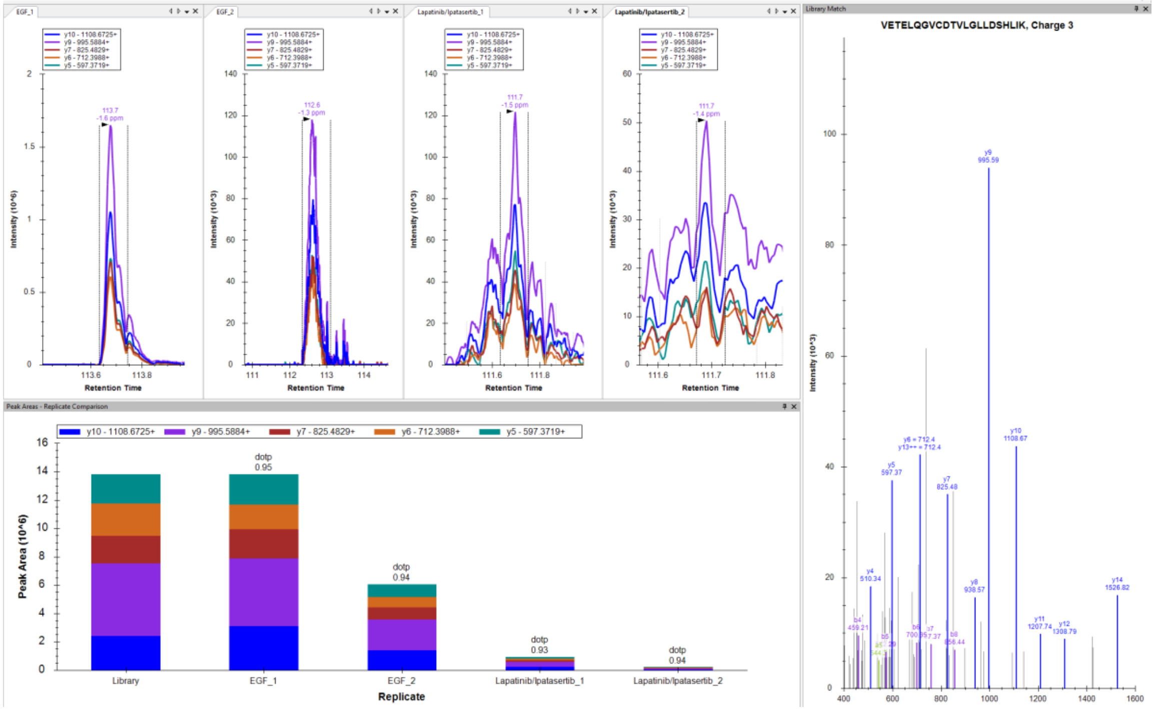

CD44 antigen (CD44)

YGFIEGHVVIPR, Charge +3, m/z = 462.9225 Da

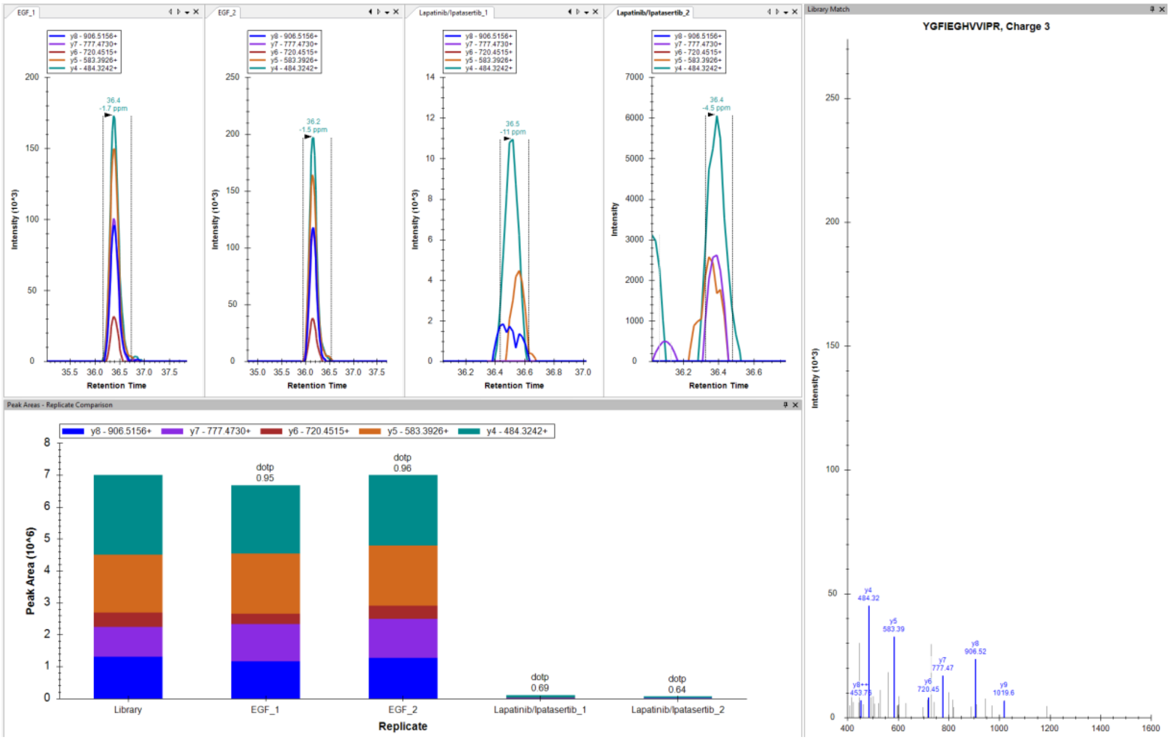

Supplement: Supplementary file 1 [file DataSheet1.zip › Supplemental file 6.PDF]
