# Supplementary material for: Proteomic assessment of SKBR3/HER2+ breast cancer cellular response to Lapatinib and investigational Ipatasertib kinase inhibitors
Source: Front Pharmacol. 2024 Aug 29;15:1413818. doi: 10.3389/fphar.2024.1413818 (PMC11391243; doi:10.3389/fphar.2024.1413818)

Supplemental file 7

1. Western blot validation of signaling inhibition by Lapatinib by assessing EGFR, ERBB2 and ERK 1/2 phosphorylation.

Protein: ERBB2/ P04626, MW = 185 kDa
Conditions:

- Primary Ab – **p-ERBB2 (Y1248)** (CST-2247) 1:1000
- Secondary Ab – Anti Rabbit HRP conjugate (CST- 7074S) 1:2000

Note: L-lapatinib

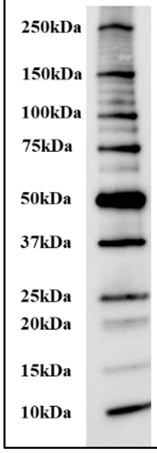

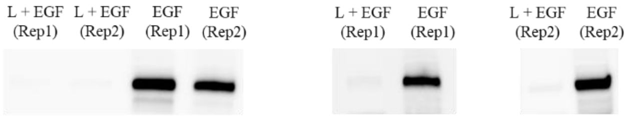

Control
EGF 60 ng/mL (15 min)

Treatment
Lap 10 uM (1 h) inhibition, followed by:
Lap 10 uM + EGF 60 ng/mL (15 min)

Control
EGF 60 ng/mL (5 min)

Treatment
Lap 10 uM (15 min) inhibition, followed by:
Lap 10 uM + EGF 60 ng/mL (5 min)

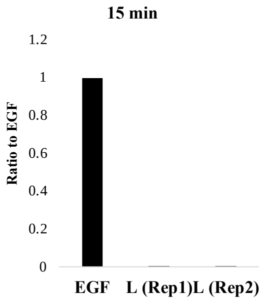

Protein: EGFR/ P00533, MW = 175 kDa
Conditions:

- Primary Ab – **p-EGFR (Y1068)** (CST-3777) on the left and **p-EGFR (Y1173)** (CST-4407) on the right 1:1000
- Secondary Ab – Anti Rabbit HRP conjugate (CST- 7074S) 1:2000

Note: L-lapatinib

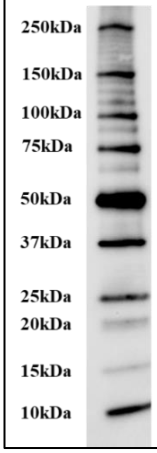

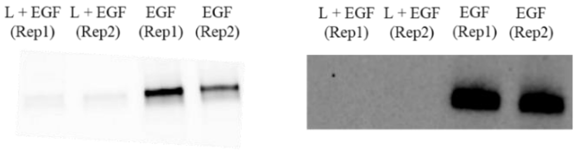

Control
EGF 60 ng/mL (15 min)

Treatment
Lap 10 uM (1 h) inhibition, followed by:
Lap 10 uM (1 h) + EGF 60 ng/mL (15 min)

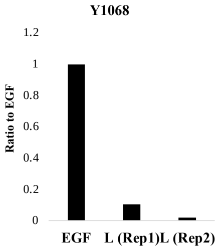

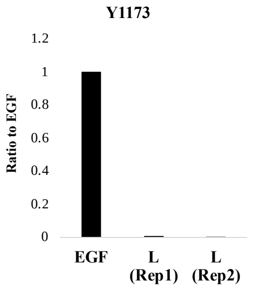

Protein: ERK1,2/ P28482, P27361 MW = 42, 44 kDa
Conditions:

- Primary Ab –**p-ERK (T202/Y204)** (CST-9101) 1:1000
- Secondary Ab – Anti Rabbit HRP conjugate (CST- 7074S) 1:2000

Note: L-lapatinib

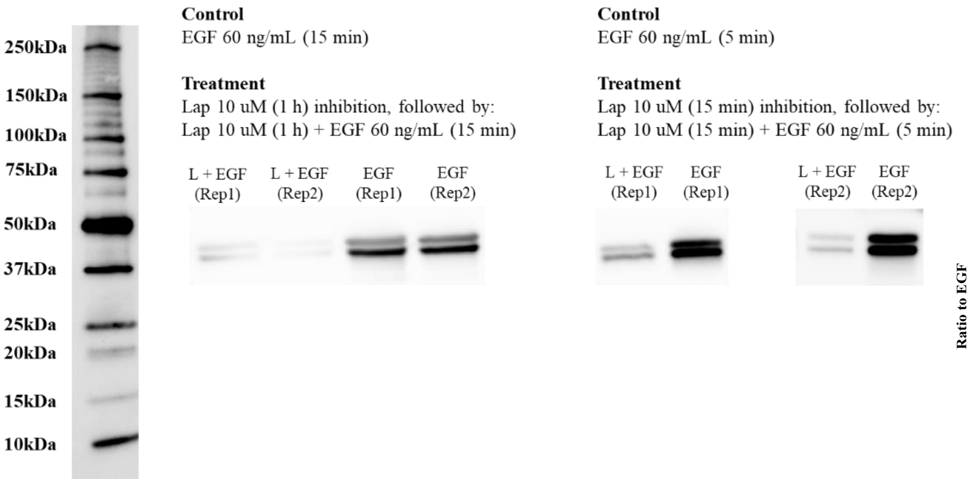

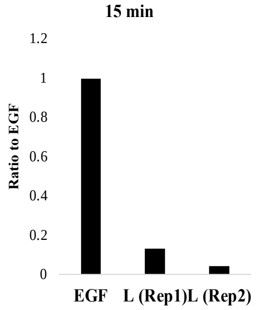

2. Western blot validation of selected proteins that changed expression level in response to the drug treatments.

Protein: VTCN1/ B7-H4/ Q7Z7D3, MW = 75 kDa, upregulated in L vs EGF and L/I vs EGF (cytoplasmic fraction)
Conditions:

- Primary Ab –VTCN1 (CST-14572) 1:1000
- Secondary Ab – Anti Rabbit HRP conjugate (CST- 7074S) 1:2000

Note: L-lapatinib, I-Ipatasertib

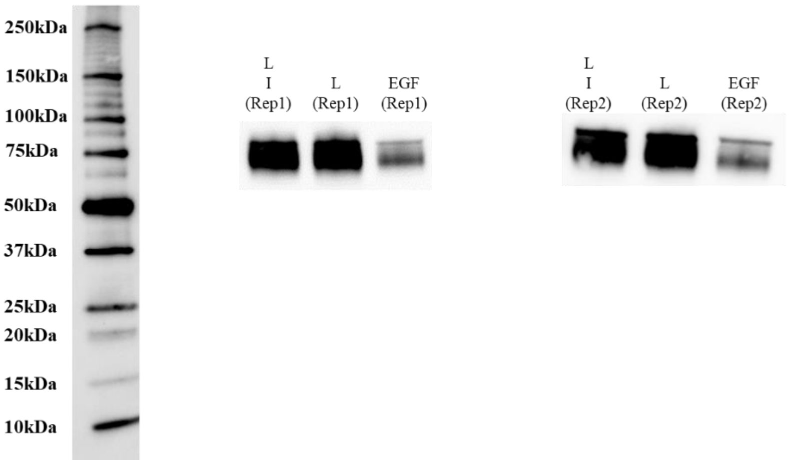

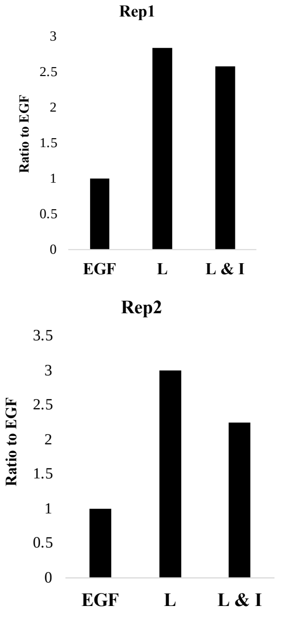

Protein: PDCD4/ Q53EL6, MW = 60 kDa, upregulated in L vs EGF and L/I vs EGF (cytoplasmic fraction)
Conditions:

- Primary Ab –PDCD4 (CST-9535) 1:1000
- Secondary Ab – Anti Rabbit HRP conjugate (CST- 7074S) 1:2000

Note: L-lapatinib, I-Ipatasertib

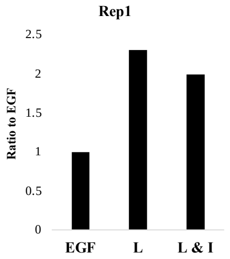

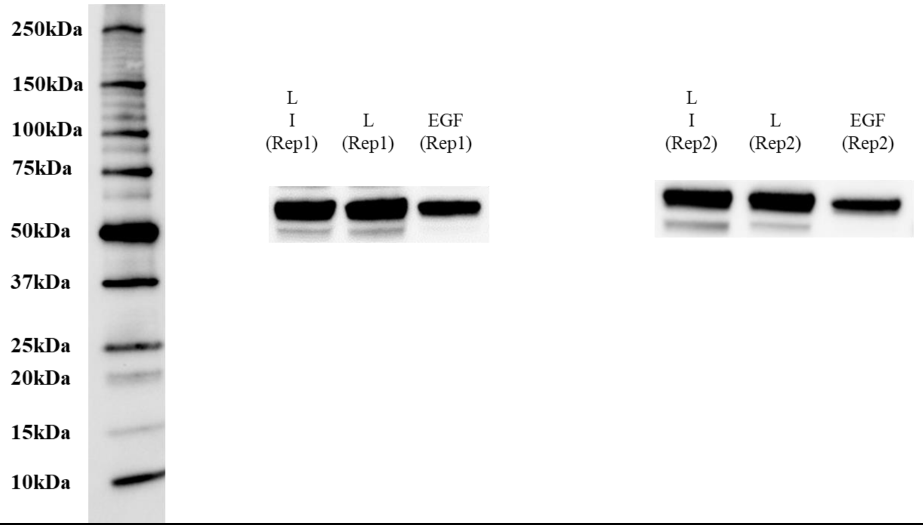

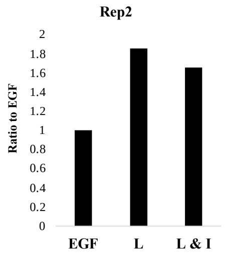

Protein: CD82/ P27701, MW = 30-90 kDa, upregulated in L vs EGF and L/I vs EGF (cytoplasmic fraction)
Conditions:

- Primary Ab –CD82 (CST-12439) 1:1000
- Secondary Ab – Anti Rabbit HRP conjugate (CST- 7074S) 1:2000

Note: L-lapatinib, I-Ipatasertib

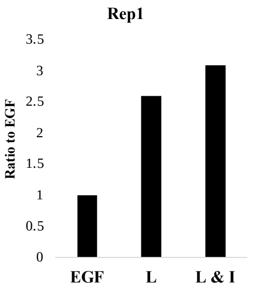

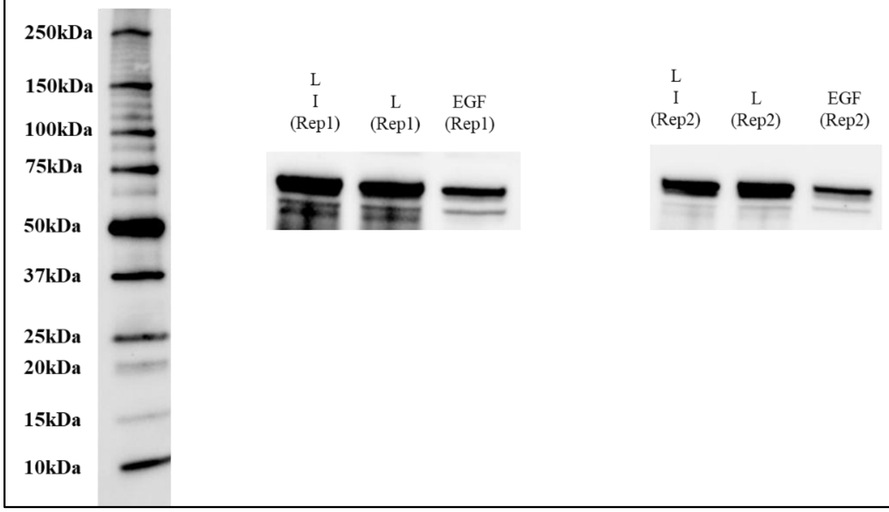

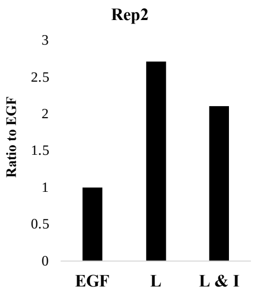

Protein: 14-3-3 sigma/ P31947, MW = 28 kDa, downregulated in L vs EGF and L/I vs EGF (cytoplasmic fraction)
Conditions:

- Primary Ab –14-3-3 sigma (RD-AF4424) 1:200
- Secondary Ab – Anti Goat HRP conjugate (RD- HAF017) 1:1000

Note: L-lapatinib, I-Ipatasertib

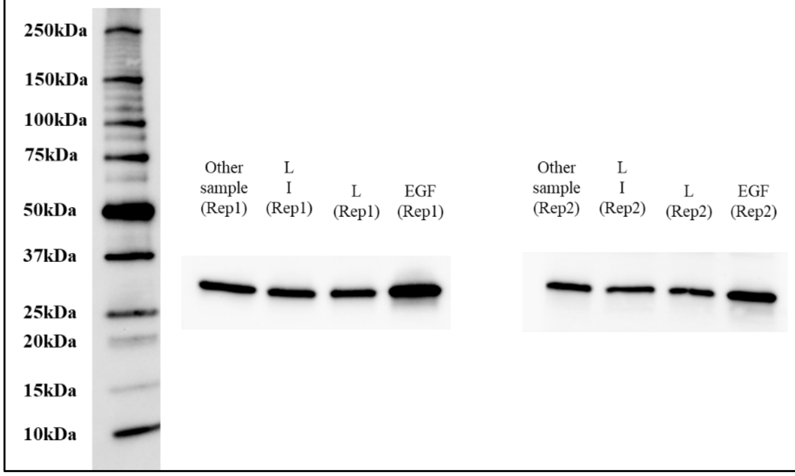

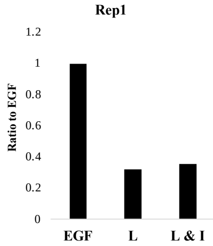

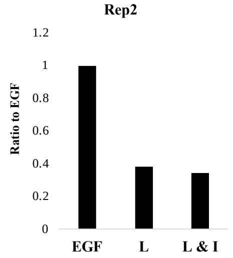

Protein: TOP2A/ P11388, MW = 190 kDa, downregulated in L vs EGF and L/I vs EGF (nuclear fraction)
Conditions:

- Primary Ab – TOP2A (CST- 12286) 1:1000
- Secondary Ab – Anti Rabbit HRP conjugate (CST- 7074S) 1:2000

Note: L-lapatinib, I-Ipatasertib

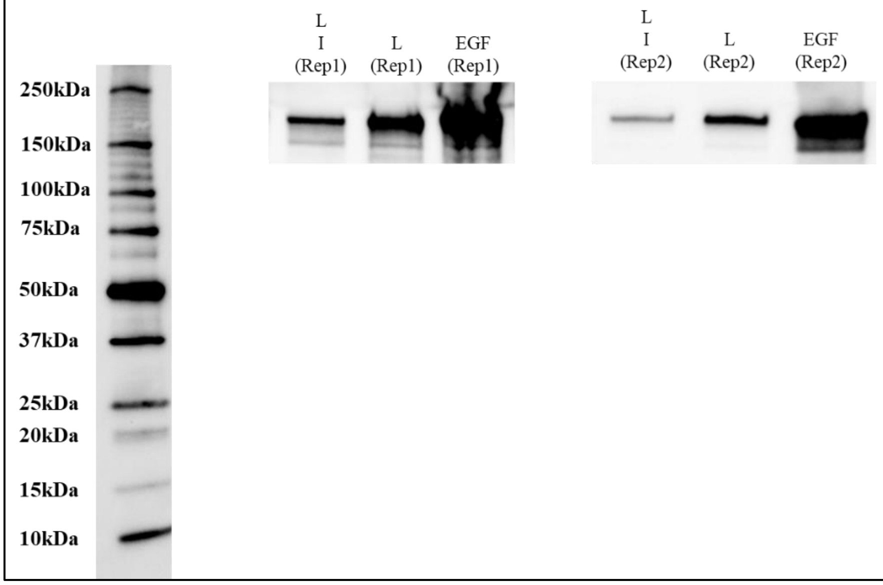

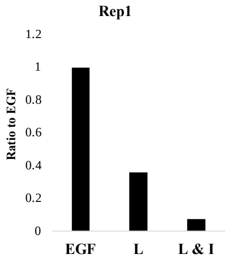

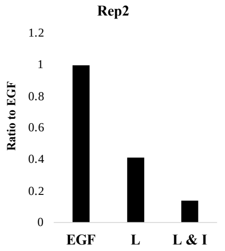

3. Full Western Blot images

p-ERBB2 Y1248 **(MW = 185 kDa)**
5 min treatment

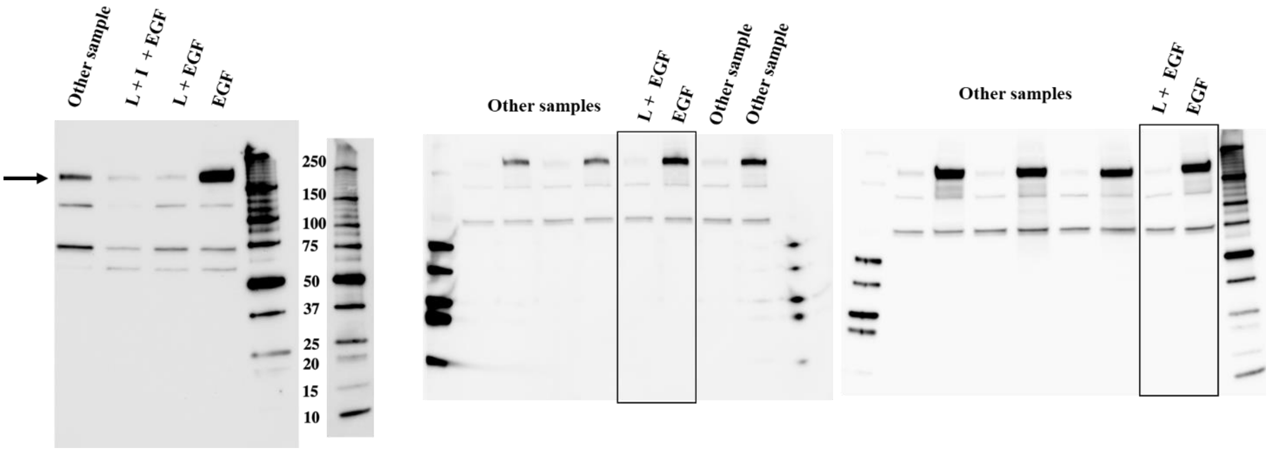

p-ERBB2 Y1248 **(MW = 185 kDa)**
15 min treatment

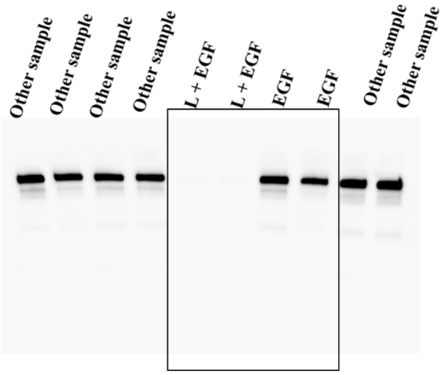

p-EGFR (Y1068) (MW = 175 kDa)
15 min treatment

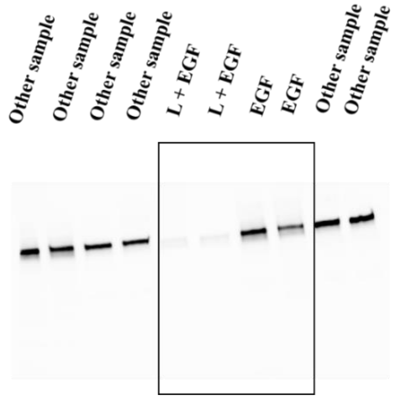

p-EGFR Y1173 **(MW = 175 kDa kDa)**
15 min treatment

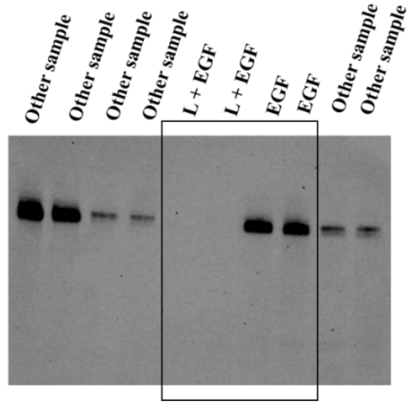

p-ERK T202/Y204 **(ERK1/ERK2 MW = 42/44 kDa)**

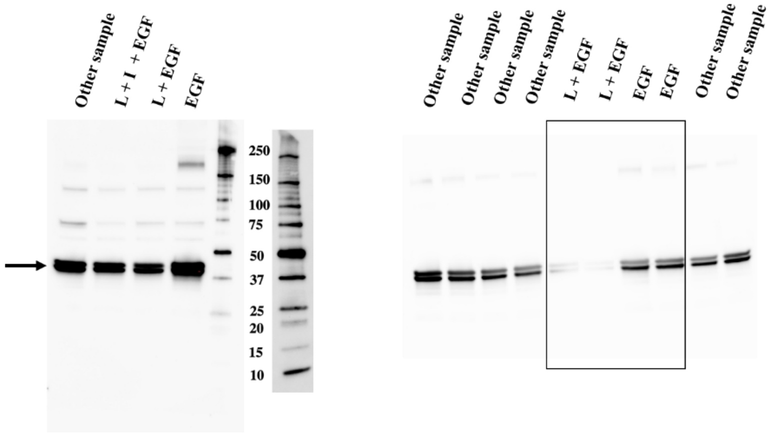

p-ERK T202/Y204 **(ERK1/ERK2 MW = 42/44 kDa)**

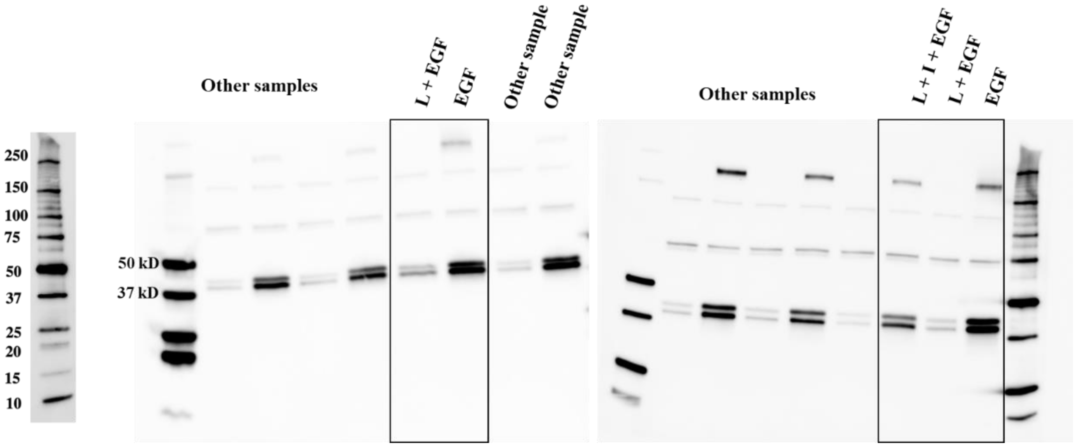

VTCN1 (B7-H4) (MW = 75 kDa)

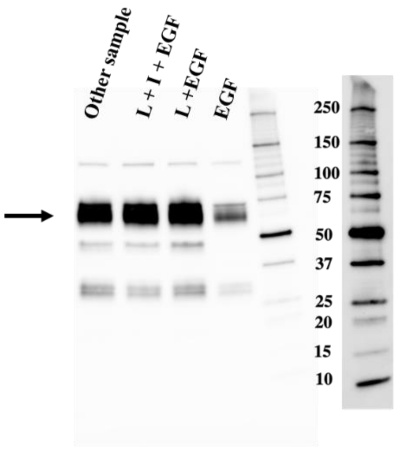

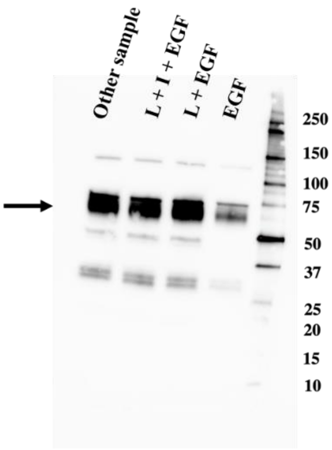

PDCD4 (MW = 60 kDa)

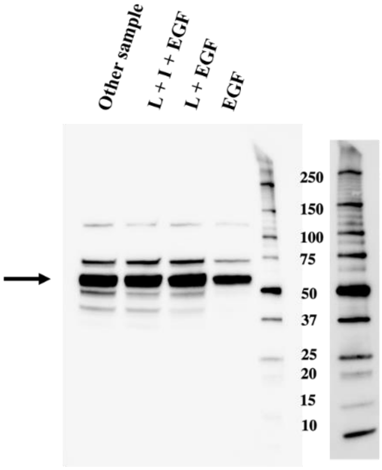

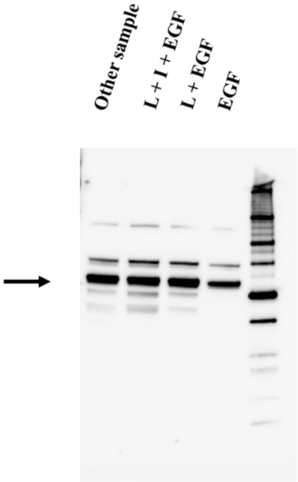

CD82 (MW = 30-90 kDa)

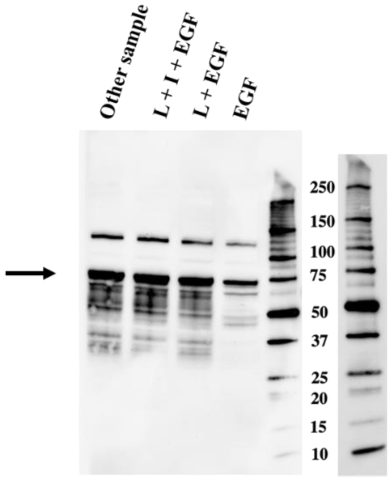

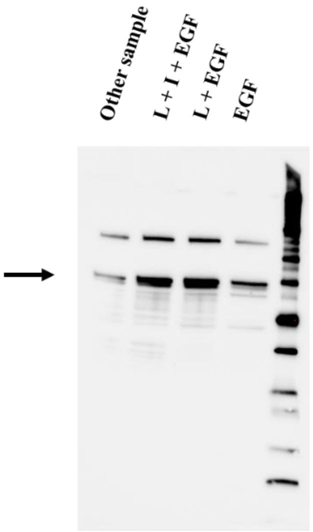

14-3-3 sigma (MW = 28 kDa)

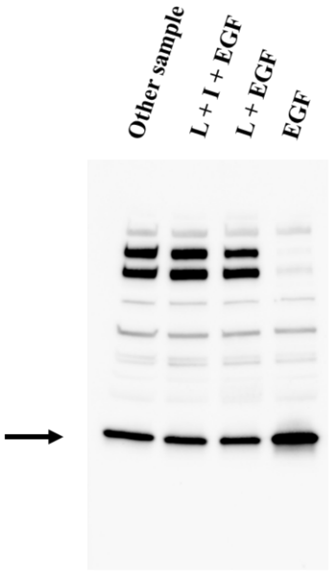

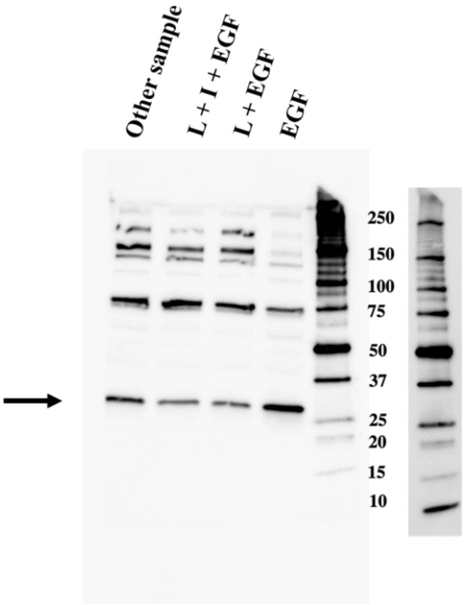

TOP2a (MW = 190 kDa)

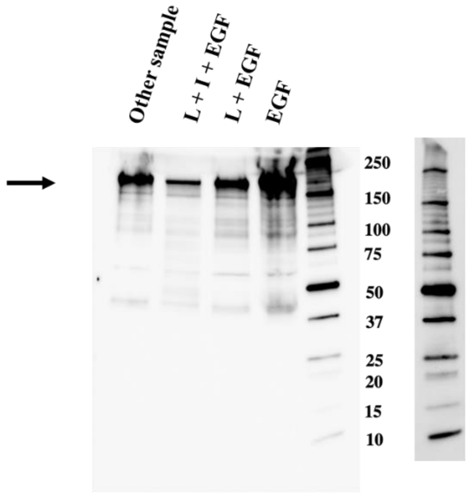

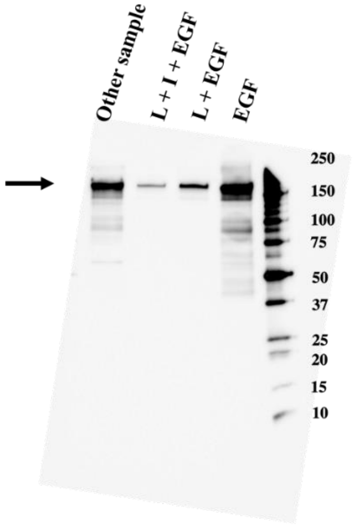

Supplement: Supplementary file 1 [file DataSheet1.zip › Supplemental file 7.PDF]
